# Blockade of integrin signaling reduces chemotherapy-induced premature senescence in collagen cultured bladder cancer cells

**DOI:** 10.1093/pcmedi/pbac007

**Published:** 2022-03-17

**Authors:** Linghui Deng, Kun Jin, Xianghong Zhou, Zilong Zhang, Liming Ge, Xingyu Xiong, Xingyang Su, Di Jin, Qiming Yuan, Chichen Zhang, Yifan Li, Haochen Zhao, Qiang Wei, Lu Yang, Shi Qiu

**Affiliations:** National Clinical Research Center of Geriatrics, the Center of Gerontology and Geriatrics, West China Hospital, Sichuan University, Chengdu 610041, China; Department of Urology, Institute of Urology and National Clinical Research Center for Geriatrics, West China Hospital, Sichuan University, Chengdu 610041, China; West China School of Medicine, Sichuan University, Chengdu 610041, China; Department of Urology, Institute of Urology and National Clinical Research Center for Geriatrics, West China Hospital, Sichuan University, Chengdu 610041, China; Department of Pharmaceutical and Bioengineering, School of Chemical Engineering, Sichuan University, Chengdu 610065, China; Department of Urology, Institute of Urology and National Clinical Research Center for Geriatrics, West China Hospital, Sichuan University, Chengdu 610041, China; Department of Urology, Institute of Urology and National Clinical Research Center for Geriatrics, West China Hospital, Sichuan University, Chengdu 610041, China; Department of Urology, Institute of Urology and National Clinical Research Center for Geriatrics, West China Hospital, Sichuan University, Chengdu 610041, China; Department of Urology, Institute of Urology and National Clinical Research Center for Geriatrics, West China Hospital, Sichuan University, Chengdu 610041, China; Department of Urology, Institute of Urology and National Clinical Research Center for Geriatrics, West China Hospital, Sichuan University, Chengdu 610041, China; Department of Urology, Institute of Urology and National Clinical Research Center for Geriatrics, West China Hospital, Sichuan University, Chengdu 610041, China; Department of Urology, Institute of Urology and National Clinical Research Center for Geriatrics, West China Hospital, Sichuan University, Chengdu 610041, China; Department of Urology, Institute of Urology and National Clinical Research Center for Geriatrics, West China Hospital, Sichuan University, Chengdu 610041, China; Department of Urology, Institute of Urology and National Clinical Research Center for Geriatrics, West China Hospital, Sichuan University, Chengdu 610041, China; National Clinical Research Center of Geriatrics, the Center of Gerontology and Geriatrics, West China Hospital, Sichuan University, Chengdu 610041, China; Department of Urology, Institute of Urology and National Clinical Research Center for Geriatrics, West China Hospital, Sichuan University, Chengdu 610041, China; Department of Molecular Oncology, Institute of Oncology Research (IOR), Oncology Institute of Southern Switzerland (IOSI), Bellinzona 6500, Switzerland

**Keywords:** premature senescence, integrins, bladder cancer, chemotherapy, collagen

## Abstract

**Background:**

Diminished sensitivity towards chemotherapy remains the major impediment to the clinical treatment of bladder cancer. However, the critical elements in control of chemotherapy resistance remain obscure.

**Methods:**

We adopted improved collagen gels and performed cytotoxicity analysis of doxorubicin (DOX) and mitomycin C (MMC) of bladder cancer cells in a 3D culture system. We then detected the expression of multidrug resistant gene *ABCB1*, dormancy-associated functional protein chicken ovalbumin upstream-transcription factor 1 (COUPTF1), cell proliferation marker Ki-67, and cellular senescence marker senescence-associated β-galactosidase (SA-β-Gal) in these cells. We further tested the effects of integrin blockade or protein kinase B (AKT) inhibitor on the senescent state of bladder cancer. Also, we examined the tumor growth and survival time of bladder cancer mouse models given the combination treatment of chemotherapeutic agents and integrin α2β1 ligand peptide TFA (TFA).

**Results:**

Collagen gels played a repressive role in bladder cancer cell apoptosis induced by DOX and MMC. In mechanism, collagen activated the integrin β1/AKT cascade to drive bladder cancer cells into a premature senescence state via the p53/p21 pathway, thus attenuating chemotherapy-induced apoptosis. In addition, TFA had the ability to mediate the switch from senescence to apoptosis of bladder cancer cells in xenograft mice. Meanwhile, TFA combined with chemotherapeutic drugs produced a substantial suppression of tumor growth as well as an extension of survival time *in vivo*.

**Conclusions:**

Based on our finding that integrin β1/AKT acted primarily to impart premature senescence to bladder cancer cells cultured in collagen gel, we suggest that integrin β1 might be a feasible target for bladder cancer eradication.

## Introduction

Bladder cancer is among the major sources of cancer-related morbidity, accounting for ∼200 000 deaths worldwide in 2018.^1^ Cytotoxic chemotherapy has been the systemic management method for bladder cancer for decades, but its efficacy remains limited.^2^ The lack of response to chemotherapy is seen as a major culprit for treatment failure in bladder cancer patients, leading to disease relapse and poor overall survival.^3^ Therefore, it is desirable to unravel the underlying mechanisms of drug resistance with the aim of devising potential treatment strategies.

Cancer cells could seek refuge from cytotoxic effects in a wide range of ways, among which the most straightforward method is to restrict drugs reaching the site of action.^4^ One such mechanism is through the upregulation of the adenosine triphosphate (ATP)-binding cassette transporters, which are able to promote drug expulsion.^5^ DNA damage repair is also considered a key player in modifying the response to chemotherapy, which may be attributed to the induction of cell cycle arrest.^6^ In addition, accumulating evidence suggested that the tumor microenvironment (TME) provided shelter for cancer cells from cytotoxic stress, leading to the development of acquired resistance.^7^ The TME is mainly made up of stromal cells and extracellular matrix (ECM) components. It has been demonstrated that collagen, the most abundant ECM protein, exerts modulatory functions in chemotherapeutic sensitivity, which may be partially dependent on cellular adhesion.^8^, ^9^ Integrins, a large family of heterodimeric receptors, serve as cell surface adhesion molecules that connect cells to the ECM. Ligand binding to integrins modulates multiple downstream signals, including the phosphoinositide 3-kinase (PI3K)–AKT, extracellular-signal regulated kinase (ERK), and NF-κB pathways, culminating in the formation of a resistant genotype.^10^ A recent study indicated that the interaction of integrin β_1_ (ITGB1) with collagen I confers chemoresistance on breast cancer cells, and ITGB1 inhibition sensitized the cells to drugs.^11^ Nevertheless, the role of integrins in chemoresistance development and the underlying mechanism remain a matter for further elucidation.

Our previous study reported that a novel 3D collagen I gel model could confer tumorigenic potential to bladder cancer cells and enable them to progress to cancer stem cells (CSCs).^12^ Herein, we further focused on the impact of collagen on chemotherapeutic sensitivity and found that collagen culture rendered bladder cancer cells into a senescent state to evade apoptosis induced by cytotoxic reagents. In mechanism, collagen regulated the integrin β1/AKT axis to trigger premature senescence, which was dependent on the p53/p21 pathway. Additionally, we confirmed that interruption of integrin signals had an inhibitory effect on chemotherapy-induced senescence *in vivo*, resulting in enhanced antitumor efficacy. Taken together, our study indicated the importance of cell senescence during integrin-induced chemoresistance development, which may aid in the elaboration of therapeutic strategies for eliminating bladder cancer.

## Methods

### Ethical approval

The clinical experiments were carried out according to the guidance of the Declaration of Helsinki and approved by the Ethics Committee of West China Hospital, Sichuan University. All patients were informed and provided written consent to participate in the study. The animal studies were conducted in accordance with the Public Health Service Policy and complied with the World Health Organization (WHO) guidelines for the humane use and care of animals. All animal protocols were monitored by the Ethics Committee of West China Hospital, Sichuan University and conducted in accordance with the ARRIVE guidelines (Animal Research: Reporting of *In Vivo* Experiments).

### Cell culture and reagents

Human bladder cancer cell line T24 was purchased from the American Type Culture Collection and maintained in RPMI 1640 complete medium (Gibco, MA, USA) supplemented with 10% fetal calf serum (Gibco, MA, USA), at 37°C in a 5% CO_2_ atmosphere. 3D collagen culture of cancer cells was performed as previously described. Briefly, collagen I was diluted to 0.6 mg/ml with RPMI 1640 complete medium supplemented with 10% fetal calf serum. Cancer cells (1 × 10^4^) were added into the 295 μl collagen solution containing 25 μl of 10× PBS and 20 μl of 1 N NaOH. After 2 h of 37°C incubation, the solid clotty collagen (containing tumor cells) was added to RPMI 1640 complete medium supplemented with 10% fetal calf serum for further use. Collagen I and type I collagenase were purchased from Sigma (USA). Integrin α2β1 ligand peptide TFA (TFA) and AKT inhibitor Miransertib (Mir) were purchased from MedChemExpress (USA). Chemotherapeutic doxorubicin (DOX) and mitomycin C (MMC) were purchased from Sangon (China).

### Primary tumor cell culture and patients’ information

Human bladder tumor tissues were collected from the West China Hospital, Sichuan University. A total of 20 tumor tissues (paraffin sections) were divided into recurrent (R) and non-recurrent (NR) groups according to a follow-up visit after chemotherapy. For establishment of primary bladder cancer cell lines, 15 primary tumor tissues (BP 1–15) were collected, and digested to be seeded in 3D Matrigel containing growth factors (#354234, Coring, USA). Two samples (BP5 and BP7) succeeded in forming colonies, which were collected and digested to seed in Matrigel again. After being cultured in Matrigel for 2 weeks, BP5 and BP7 were digested and cultured in a dish for further analysis. Both BP5 and BP7 survived and displayed proliferative characteristics in the dish for 4 months.

### Cell apoptosis analysis

Cell apoptosis was determined using the FITC-Annexin V and PE-PI apoptosis detection kit (Becton, Dickinson and Company, USA). Briefly, pre-treated tumor cells were harvested and stained with FITC-Annexin V and PE-PI viability staining solution for 15 min at room temperature. Subsequently, apoptosis was analyzed by flow cytometry on a C6 flow cytometer (Becton, Dickinson and Company, USA).

### Senescence-associated β-galactosidase assay

Cells were stained for β-galactosidase (β-Gal) activity according to the guidance of the senescence-associated β-Gal (SA-β-Gal) assay kit (Biyuntian, China). Briefly, tumor cells were digested and washed in phosphate-buffered saline (PBS), then fixed for 10 min in 4% formaldehyde. After that, tumor cells were incubated at 37°C (containing no CO_2_) with staining solution (1 mg/ml 5-bromo-4-chloro-3-indolyl β-D-galactoside). After 12 h, quantification of SA-β-Gal-positive cells was performed under a photon microscope (Leica, Germany).

### Cell cycle assay

Cell cycle analysis was performed using the PE-PI cell cycle analysis kit (Becton, Dickinson and Company, USA). In brief, pre-treated cancer cells were harvested and fixed with cold ethanol. Subsequently, samples were incubated with propidium iodide (PI) staining solution for 10 min according to the manufacturer's protocol. The samples were detected by flow cytometry (Becton, Dickinson and Company, USA).

### Real-time quantified polymerase chain reaction

The quantification of mRNA levels was conducted by real-time polymerase chain reaction (PCR) using SYBR green dye (Thermo, MA, USA). *GAPDH* was used for normalization. The primers used were as follows: human *GAPDH* forward primer 5′-GGAGCGAGATCCCTCCAAAAT-3′ and reverse primer 5′-GGCTGTTGTCATACTTCTCATGG-3′; human *COL1A1* forward primer 5′- GAGGGCCAAGACGAAGACATC-3′ and reverse primer 5′-CAGATCACGTCATCGCACAAC-3′; human *COL1A2* forward primer 5′-GTTGCTGCTTGCAGTAACCTT-3′ and reverse primer 5′-AGGGCCAAGTCCAACTCCTT-3′; human *ABCB1* forward primer 5′-TTGGCTGATGTTTGTGGGAAG-3′ and reverse primer 5′-CCAAAAATGAGTAGCACGCCT-3′.

### Western blotting

Cells were harvested and homogenized in lysis buffer (Sigma, USA) containing phosphatase and proteinase inhibitors (Sigma, USA). Protein samples were then quantified using the Pierce BCA Protein Assay (Thermo Fisher, USA). Protein samples (25 μg) were separated by SDS-PAGE, transferred to PVDF membranes, and incubated with primary antibodies for integrin β1 (ITGB1) (1:1000; Abcam, B179471, UK), p21 (1:1000; Abcam, ab109199, UK), p53 (1:1000; Abcam, ab32389, UK), total AKT (1:1000; Abcam, ab8805, UK), p-AKT (1:500; Abcam, ab38449, UK), or β-actin (1:1000; Abcam, ab8226, UK) at 4°C overnight. Bound antibodies were detected by horseradish peroxidase-linked secondary antibodies (Thermo Fisher, USA) and processed with Pierce ECL Western Blotting Substrate (Thermo Fisher, USA).

### Immunofluorescence and immunohistochemistry

Tumor tissues were fixed in 10% formalin solution for at least 48 h. Subsequently, samples were processed, embedded in paraffin, and sectioned at 5 μm for immunofluorescence and immunohistochemical staining. Sections were then dewaxed, rehydrated, quenched of endogenous peroxidase, blocked, and incubated with the primary antibody anti-collagen I (1:100; Abcam, ab270993, UK) at 4°C overnight, followed by signal amplification using horseradish peroxidase-linked secondary antibodies (Thermo Fisher, USA). The intensity of collagen I expression was determined by Image-Pro Plus 6.0 software (USA).

### *In vivo* experiments

Nude mice (6–8 weeks old) were purchased from Huafukang (Beijing, China) and housed in a specific pathogen-free facility. For cell senescence and apoptosis analysis *in vivo*, 5 × 10^6^ T24 cells were subcutaneously injected into nude mice. On day 20, mice were treated with PBS, MMC (5 mg/kg), or DOX (5 mg/kg) by tail vein injection (*n* = 3 in each group). On day 23, mice were sacrificed for tumor tissue collection. p21 and p53 expression were determined by western blotting. Part of the tumor tissues were digested for cell apoptosis and senescence analysis. For tumor volume and survival analysis, 5 × 10^6^ T24 cells were subcutaneously injected into nude mice. On day 20, mice were treated with PBS, MMC (5 mg/kg), TFA (2.5 mg/kg), or a combination twice a week by tail vein injection (*n* = 6 in each group). Tumor volume and survival were recorded every day. The formula for the calculation of tumor volume is: tumor volume = length × width^2^/2.

### Statistical analysis

All data are presented as the mean ± SEM. Statistical significance was analyzed using GraphPad 6.0 software (La Jolla, CA, USA). Statistical significance between groups was calculated by Student's t test for two groups or by one-way ANOVA for more than two groups. Bonferroni analysis was further used for *post hoc* testing. Survival rates were determined by Kaplan–Meier survival analysis. *P < 0.05; **P < 0.01; ns, no significant difference. Each experiment was performed at least three times, independently.

## Results

### Collagen reduced chemotherapy-induced cell apoptosis and correlated with tumor recurrence in patients

**Figure 1.**
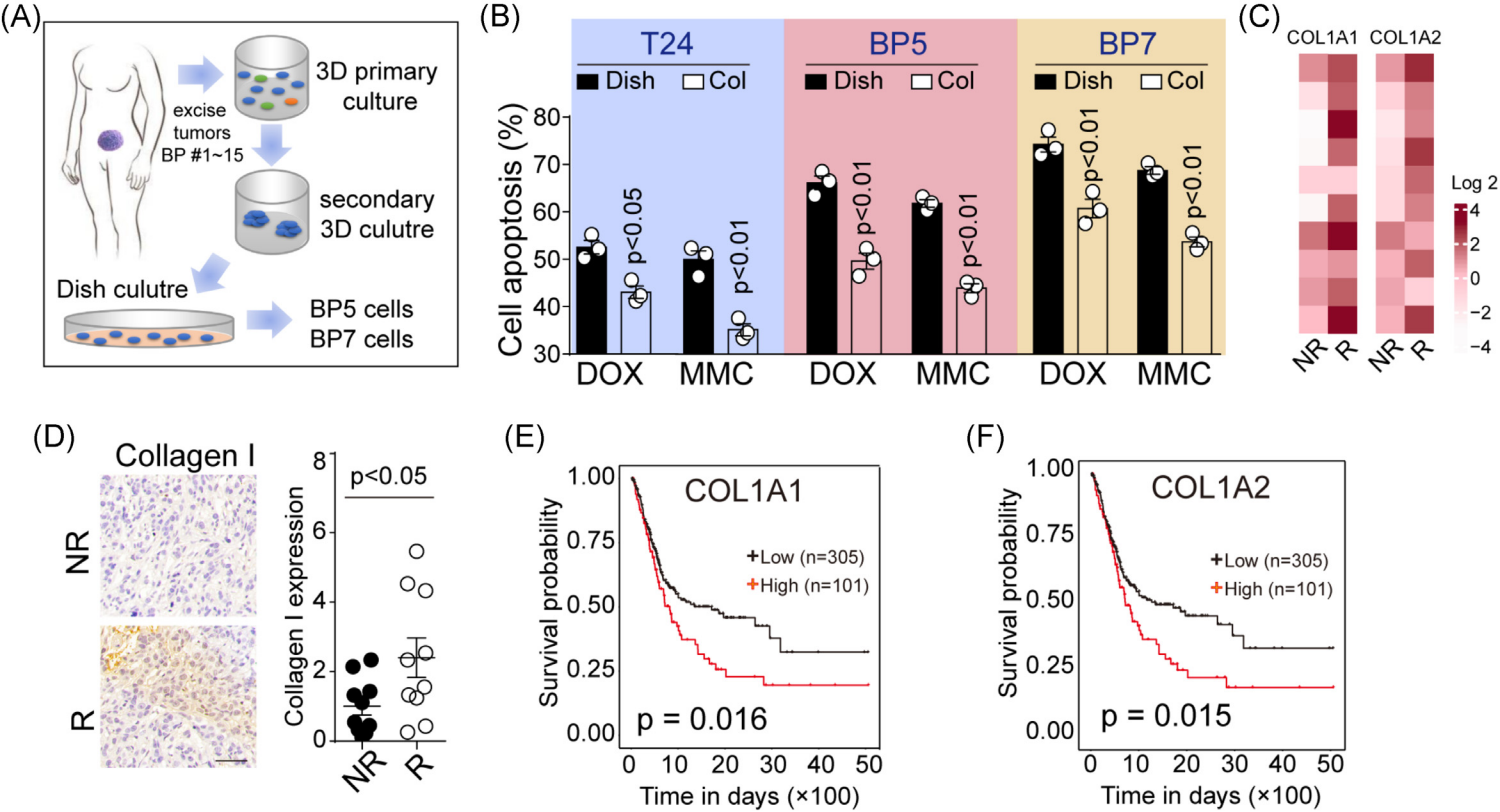
Collagen reduced chemotherapy-induced cell apoptosis and correlated with tumor recurrence in patients. (**A**) Schematic diagram of primary tumor cells culture (15 tumor tissues, BP1–15). (**B**) Cell apoptosis of T24, BP5 and BP7 (dish or collagen culture) treated with MMC (0.5 μg/ml, 48 h) or DOX (1 μg/ml, 48 h). (**C**) Relative expression of *COL1A1* and *COL1A2* at mRNA level in bladder tumor tissues from 20 patients divided into recurrent (R, *n* = 10) and non-recurrent (NR, *n* = 10) groups. (**D**) Immunohistochemical staining of collagen I in bladder tumor tissues from 20 patients divided into recurrent (R, *n* = 10) and non-recurrent (NR, *n* = 10) groups. The scale bar is 50 μm. (**E**) Overall survival analysis of bladder cancer patients with high/low level *COL1A1* expression (*n* = 406) using the TCGA database. (**F**) Overall survival analysis of bladder cancer patients with high/low level *COL1A2* expression (*n* = 406) using the TCGA database.

Our previous work has demonstrated that collagen I served as the major element in ECM and was tightly correlated with the tumorigenic potential of tumor initiating cells (TICs).^12^ Stemming from this evidence, we next sought to investigate the clinical relevance of the role of collagen in regulating chemoresistance during bladder cancer progression. To do this, 15 tumor tissues were collected from bladder cancer patients, and tumor cells were digested and seeded in 3D matrix gels (containing growth factors) for primary cancer cells culture. Two primary bladder cancer cell lines (bladder patient #5, BP5 and bladder patient #7, BP7) succeeded in surviving in the dish culture and exhibited proliferative characteristics for 4 months (Fig. 1A). Subsequently, bladder cancer cell line T24 and primary BP5/BP7 cells were seeded in our improved collagen gels as described. To verify whether collagen was able to promote chemoresistance, MMC and DOX were added into the culture medium of dish/collagen cultured bladder cancer cells (T24, BP5 and BP7), and cell apoptosis was assessed by Annexin V/PI assay after 48 h. As expected, analysis by flow cytometry showed that collagen cultured bladder cancer cells (T24, BP5 and BP7) were significantly resistant to DOX (Fig. 1B) when compared to the dish cultured group. Notably, enhanced resistance to MMC was observed in collagen cultured cells (Fig. 1B), suggesting that *in vitro* 3D collagen culture reduced the cytotoxicity of chemotherapy in bladder cancer cells. Seeking to further assess the clinical relationship between collagen I and chemoresistance development, we acquired information and tumor specimens of 20 bladder cancer patients, and then divided these patients into two groups, including NR and R tumor groups, according to a follow-up visit after standard surgery combing adjuvant chemotherapy. Here, the expression of collagen I associated genes (*COL1A1* and *COL1A2*) in the two groups was analyzed by quantified PCR. Of note, we observed that the R group displayed elevated expression of *COL1A1* and *COL1A2* when compared to the NR group (Fig. 1C). Consistently, the protein expression of collagen I in the R group was higher than in the NR tumors (Fig. 1D). These data suggested that elevated collagen I expression might be the causative reason for therapeutic resistance and tumor relapse. Additionally, we assessed the overall survival of bladder cancer patients with high/low *COL1A1/COL1A2* expression by analyzing the Cancer Genome Atlas Program (TCGA) databases. Notably, patients with low levels of *COL1A1/COL1A2* expression had a remarkably longer survival time than those with high levels (Fig. 1E and F). Together, these results suggested that collagen reduced chemotherapy-induced cell apoptosis and correlated with tumor recurrence and poor prognosis in bladder cancer patients.

### Chemotherapy-induced cellular senescence to escape apoptosis in 3D collagen cultured TICs

**Figure 2.**
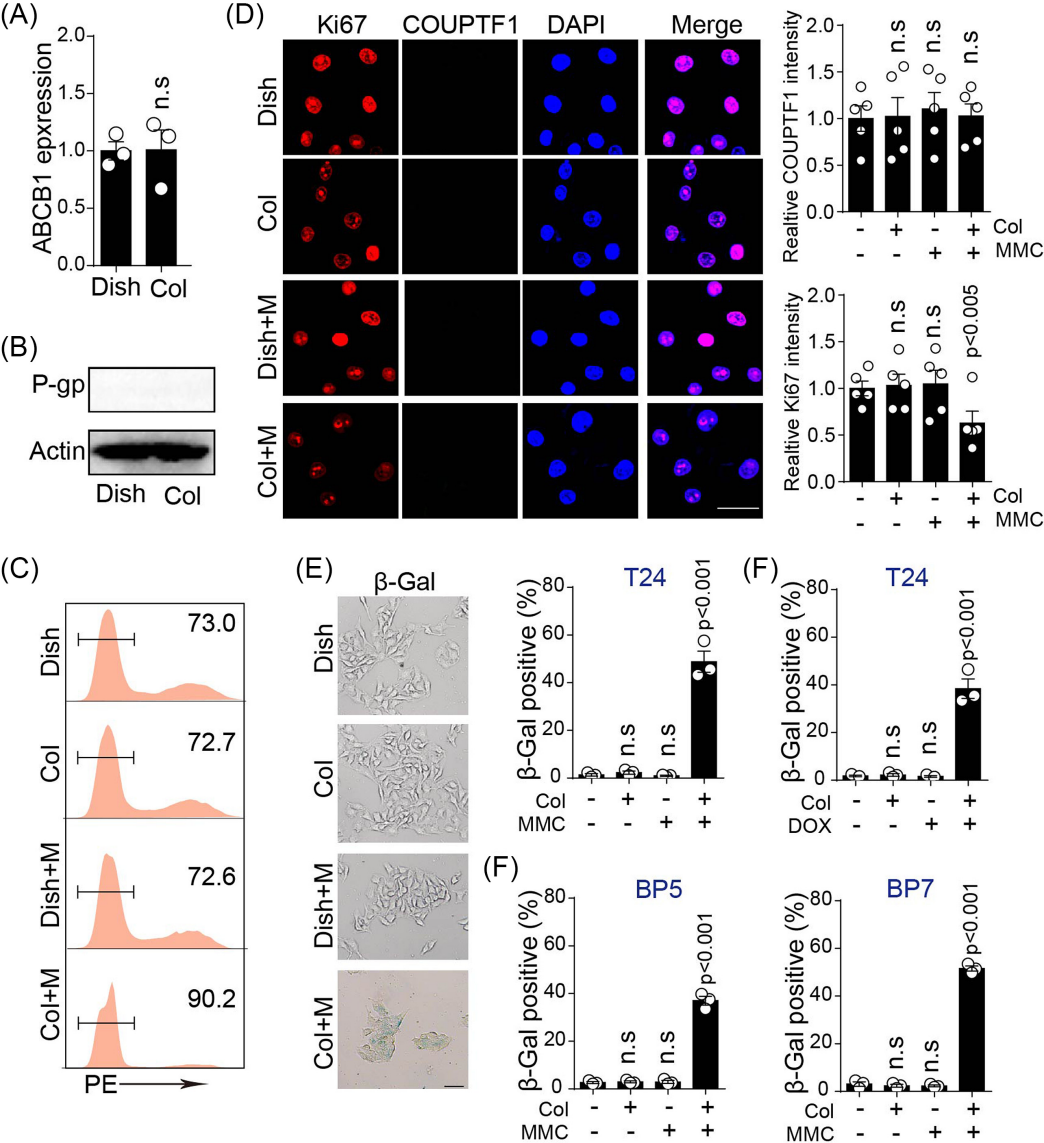
Chemotherapy induced cellular senescence to escape apoptosis in 3D collagen cultured TICs. (**A**) Relative *ABCB1* expression at mRNA level in T24 cells cultured in a dish or collagen gel. (**B**) Western blotting of P-glycoprotein (P-gp) in T24 cells cultured in a dish or collagen gel. (**C**) Cell cycle analysis of T24 cells (cultured in a dish or collagen gel) treated with PBS or MMC (0.5 μg/ml, 48 h), using PI staining. (**D**) Immunofluorescence of Ki67 and COUPTF1 in T24 cells (cultured in a dish or collagen gel) treated with PBS or MMC (0.5 μg/ml, 48 h); the scale bar is 30 μm. Fluorescence intensity was determined by Image J 6.0 software. (**E**) SA-β-Gal staining of T24 cells (cultured in a dish or collagen gel) treated with PBS or MMC (0.5 μg/ml, 48 h); the scale bar is 30 μm. (**F**) SA-β-Gal-positive cells analysis of T24 cells (cultured in a dish or collagen gel) treated with PBS or DOX (1 μg/ml, 48 h). (**G**) SA-β-Gal-positive cells analysis of BP5 and BP7 cells (cultured in a dish or collagen gel) treated with PBS or MMC (0.5 μg/ml, 48 h).

Motivated by prior results implying that collagen reduced cell cytotoxicity induced by chemotherapeutic agents, we next sought to elucidate the mechanism underlying collagen-induced chemoresistance. Development of chemoresistance is bound up with diverse biological processes, including upregulation of multidrug resistant protein P-glycoprotein and arrest of cell cycle (cell dormancy and senescence).^13^ We first examined the expression of multidrug resistant gene *ABCB1* in dish- and collagen/MMC-cultured T24 cells by quantified PCR, respectively. However, no significant alterations in gene expression were detected for *ABCB1* in bladder cancer cells (Fig. 2A). Similarly, we did not find upregulation of multidrug resistant protein P-glycoprotein at the protein level in collagen/MMC-cultured cancer cells when compared to the dish group (Fig. 2B). These results implied that collagen reduced chemotherapy-induced cell apoptosis in a multidrug resistant proteins-independent manner. Subsequently, we wondered whether collagen played a role in regulating the cell cycle to help tumor cells escape from apoptosis. Thus, we assessed the cell cycle of dish- and collagen-cultured bladder cancer cells using AV-PI staining, respectively. Intriguingly, no significant difference was observed in cell cycle between dish- and collagen-cultured T24 cells, while MMC-treated T24 cells (collagen culture) were arrested in the G0/G1 phase (Fig. 2C), suggesting that chemotherapy combining collagen culture induced cell cycle arrest, thus affecting cell apoptosis. As previously reported, a senescence or dormancy-like state could enable tumor cells to be arrested in the G0/G1 state, which would help tumor cells to escape the apoptosis state during chemotherapy. Thus, to assess whether collagen-cultured bladder cancer cells entered a dormancy-like state, an immunofluorescence assay was performed to examine dormancy-associated functional protein chicken ovalbumin upstream-transcription factor 1 (COUPTF1) and cell proliferation marker Ki-67 expression in collagen/MMC-treated T24 cells. Unfortunately, no significant difference was detected in COUPTF1 expression among groups (dish, collagen, MMC, or collagen combining MMC, Fig. 2D). This suggested to us that T24 cells might progress to a premature senescence-like state, instead of dormancy, to escape apoptosis. To test our hypothesis, an SA-β-Gal (a senescence marker) assay was conducted to evaluate cell senescence in T24 cells. Remarkably, MMC treatment enhanced SA-β-Gal expression in T24 cells (cultured in collagen), whereas the expression of SA-β-Gal was unchanged in mono MMC or collagen cultured group (Fig. 2E). Similarly, an elevated expression level of SA-β-Gal was found in DOX-treated T24 (collagen culture, Fig. 2F) and BP5/BP7 (Fig. 2G) cells. Based on these findings, it was suggested that collagen culture promoted the entry of tumor cells into a senescent state in the presence of chemotherapeutic agents. Collectively, these results suggested that collagen culture enabled bladder cancer cells to progress to a senescent state to escape apoptosis induced by chemotherapeutic MMC and DOX.

### Integrin–AKT axis induced premature senescence via p53/p21 pathway

**Figure 3.**
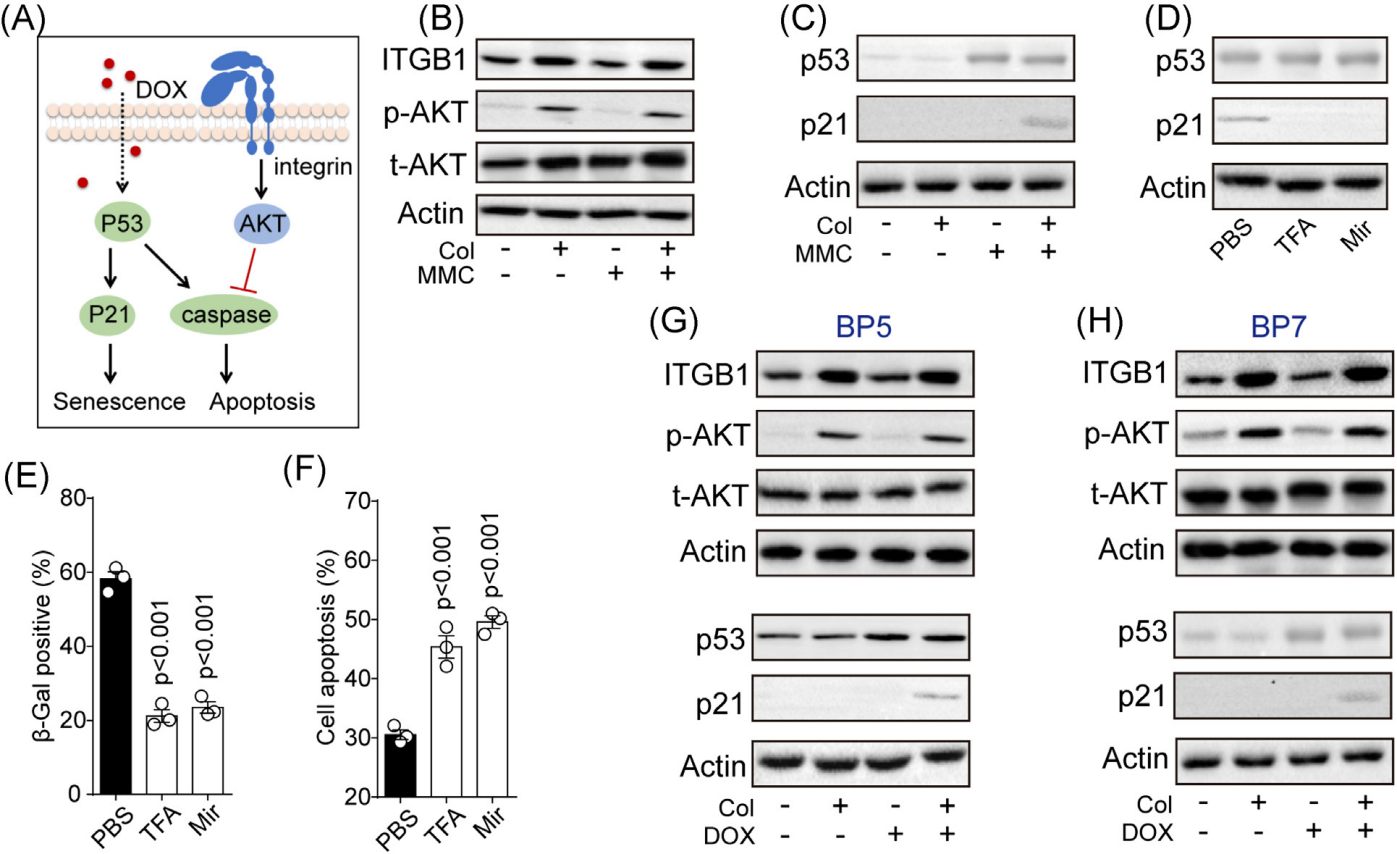
The integrin–AKT axis induced premature senescence via the p53/p21 pathway. (**A**) Schematic diagram of integrin reducing chemotherapy-induced cell apoptosis and promoting premature senescence through p53/p21 signaling. (**B**) Western blotting of ITGB1, phosphorylated AKT and total AKT in T24 cells (cultured in a dish or collagen gel) treated with PBS or MMC (0.5 μg/ml, 48 h). (**C**) Western blotting of p53 and p21 in T24 cells (cultured in a dish or collagen gel) treated with PBS or MMC (0.5 μg/ml, 48 h). (**D**) Western blotting of p53 and p21 in T24 cells (collagen culture, 0.5 μg/ml MMC treatment) pre-treated with PBS, Mir (10 nM), or TFA (1 μM). (**E**) SA-β-Gal-positive cells analysis of T24 cells (collagen culture, 0.5 μg/ml MMC treatment) pre-treated with PBS, Mir (10 nM), or TFA (1 μM). (**F**) Cell apoptosis of T24 cells (collagen culture, 0.5 μg/ml MMC treatment) pre-treated with PBS, Mir (10 nM) or TFA (1 μM). (**G**) Western blotting of ITGB1, phosphorylated AKT, total AKT, p53, and p21 in BP5 cells (cultured in a dish or collagen gel) treated with PBS or DOX (1 μg/ml, 48 h). (**H**) Western blotting of ITGB1, phosphorylated AKT, total AKT, p53, and p21 in BP7 cells (cultured in a dish or collagen gel) treated with PBS or DOX (1 μg/ml, 48 h).

Given the crucial role of collagen/chemotherapy in regulating the cell cycle, we next sought to elucidate the molecular pathway driving cell senescence. As reported in our previous study, biomaterial 3D collagen gels reverted differentiated tumor cells back into CSCs through the integrin α2β1–AKT cascade. Importantly, a compelling report provided evidence that the integrin–Src–AKT axis could mediate cellular senescence by counteracting apoptosis in irradiated tumor cells.^14^ Thus, we put forward the hypothesis that collagen induces upregulation of integrin β1, which leads to activation of the AKT signaling pathway. Subsequently, activated AKT blocks caspase activation and p21 cleavage induced by chemotherapy, eventually resulting in p53/p21 signaling activation and cell senescence, instead of apoptosis, in bladder cancer cells (Fig. 3A). To confirm this, western blotting was performed to examine the integrin β1–AKT axis in T24 cells with four process modes (dish, collagen, MMC, MMC and collagen combination). As excepted, western blotting analysis revealed that both integrin β1 and AKT were notably upregulated in collagen-cultured cells (Fig. 3B), indicating that collagen mediated integrin–AKT axis activation. *p53* is a critical tumor suppressor gene, and activating p53 and its downstream targets to induce apoptosis is a traditional apoptosis pathway in tumor cells during chemotherapy. p21 is an inhibitor of cyclin-dependent kinase, which has been reported to serve as a downstream molecule of p53 to promote cell senescence.^15^ Thus, we further examined p53/p21 expression in T24 cells (dish, collagen, MMC, MMC and collagen combination). As shown by western blotting, MMC treatment upregulated p53 expression, and collagen culture mediated p21 activation in MMC-treated T24 cells (Fig. 3C). These results implied that MMC mediated p53 apoptosis pathway activation, and collagen culture might counteract cell apoptosis and mediate p21-associated cell senescence. Subsequently, in an attempt to clarify whether the activation of p53/p21 signaling was affected by the integrin–AKT axis, T24 cells were seeded into collagen gels and treated with MMC, followed by TFA and AKT inhibitor Mir treatment. We found that blockade of integrin or AKT induced strongly repressed expression of p21, while the protein level of p53 was unchanged (Fig. 3D). Consistently, SA-β-Gal and cell apoptosis analysis suggested that TFA and Mir treatment suppressed cell senescence (Fig. 3E) and increased cell apoptosis (Fig. 3F) in T24 cells (collagen and MMC treatment). These results suggest that collagen promoted integrin–AKT axis activation to induce premature senescence via the p53/p21 pathway to escape MMC-induced cell apoptosis. To further verify our results, expression of integrin β1–AKT and p53/p21 signaling was evaluated in DOX-treated BP5 and BP7 cells. Similar results were observed in DOX-treated BP5/BP7 cells (Fig. 3G and H). Together, these results suggest that collagen regulated the integrin–AKT axis to induce premature senescence via the p53/p21 pathway.

### Blockade of integrin–ATK signaling suppressed chemotherapy-induced senescence *in vivo*

**Figure 4.**
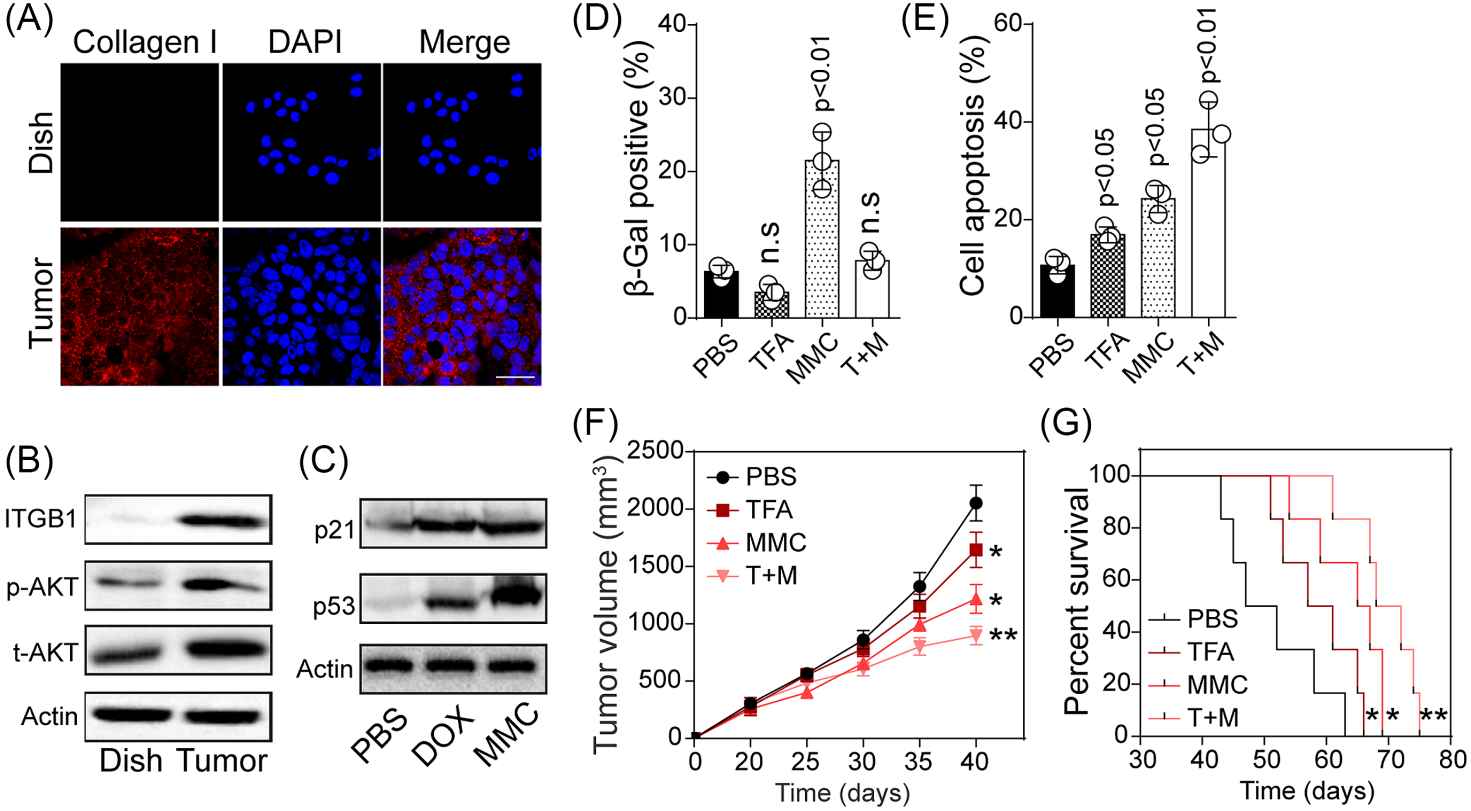
Blockade of integrin–ATK signaling suppressed chemotherapy-induced senescence *in vivo*. (**A**) Immunofluorescence of collagen in dish-cultured T24 or tumor tissues from T24-bearing mice. The scale bar is 50 μm. (**B**) Western blotting of ITGB1, phosphorylated AKT and total AKT in dish-cultured T24 or tumor tissues from T24-bearing mice. (**C**) Western blotting of p53 and p21 in tumor tissues from T24-bearing mice treated with PBS, MMC (5 mg/kg) or DOX (5 mg/kg) by tail vein injection. (**D**) SA-β-Gal-positive cells analysis in tumor tissues from T24-bearing mice treated with PBS, MMC (5 mg/kg), TFA (2.5 mg/kg) or combination. (**E**) Cell apoptosis in tumor tissues from T24-bearing mice treated with PBS, MMC (5 mg/kg), TFA (2.5 mg/kg) or combination. (**F**) Tumor volume of T24-bearing mice treated with PBS, MMC (5 mg/kg), TFA (2.5 mg/kg) or combination. (**G**) Overall survival of T24-bearing mice treated with PBS, MMC (5 mg/kg), TFA (2.5 mg/kg), or combination. n.s, no significant difference, *, *P* < 0.05, **, *P* < 0.01.

Stemming from our *in vitro* results, we next became interested in assessing the influence of senescence on chemotherapy *in vivo*. To do this, we first checked the expression of collagen–integrin β1–ATK expression in T24-bearing mice *in vivo*. In fact, collagen I, the dominant component in the ECM, has been detected in a wide range of tumor tissues. Consistent with previous reports, a higher expression of collagen I in subcutaneous T24 tumor tissues was found when compared to dish culture (Fig. 4A). Meanwhile, activation of integrin β1–AKT signaling was observed in T24 tumor tissues (Fig. 4B), which was in line with the elevated expression of collagen I *in vivo*. Next, we treated T24-bearing mice with DOX and MMC by tail vein injection. After 3 days, mice were sacrificed for tumor tissue collection and p53/p21 expression analysis. Accordingly, the protein levels of p53 and p21 were both much higher in MMC- or DOX-treated tumor tissues when compared to the PBS group (Fig. 4C). Those results were in line with the hypothesis that activation of integrin enhanced cell senescence through p53/p21 signaling in the presence of chemotherapeutic agents. Subsequently, we established a subcutaneous T24-bearing mouse model, followed by MMC and TFA treatment (day 20), for cell senescence and apoptosis assay. At 48 h later, reduced SA-β-Gal-positive cells (Fig. 4D) and enhanced cell apoptosis (Fig. 4E) were found in mice treated with MMC/TFA combination, when compared to the MMC group. Additionally, TFA and MMC combination efficiently halted tumor growth (Fig. 4F) and extended the survival time of tumor-bearing mice (Fig. 4G). These results supported the notion that blockade of integrin/ATK signaling suppressed chemotherapy-induced senescence *in vivo*, resulting in an improved tumor suppressive effect.

## Discussion

Development of chemoresistance is intimately linked with the microenvironment which comprises both neoplastic cells and stromal components. The bidirectional communications between tumor cells and the stroma are thought to initiate events that contribute to drug resistance.^16^ A large body of information exists regarding the role of collagen, the main constituent of the ECM, in modulating the response of tumors to diverse treatments. Moon *et al*. reported that ovarian cancer cells within a collagen-based hydrogel were less vulnerable to the induction of apoptosis by antitumor agents, resulting in a higher level of drug resistance.^17^ It was also demonstrated that collagen mediated tumor initiating cell-like characteristics and decreased sensitivity to 5-fluorine and paclitaxel in gastric cancer.^18^ In a study of lung carcinoma, chemo-resistant patients exhibited enhanced expression of COL1A1, which might serve as an independent prognostic factor.^19^ As for bladder cancer, we established an improved 3D culture system and found that collagen gels had an inhibitory effect on cell apoptosis induced by MMC and DOX. Clinical data further revealed elevated collagen I expression in patients with recurrent bladder cancer. Furthermore, analysis of the TCGA database correlated a survival benefit with low levels of *COL1A1/COL1A2* in bladder cancer patients. The above results highlighted that collagen promoted drug resistance in bladder cancer and was closely associated with tumor relapse.

Based on the crucial role of collagen, we sought to elucidate the specific mechanism whereby collagen suppressed chemotherapy-mediated cell apoptosis. Evidence is accumulating that apoptosis is not the only way in which tumor cells respond to drug stimuli. Tumor cells failing to undergo apoptosis may enter into a terminally arrested state termed premature senescence.^20^ A panel of markers participate in defining a cell as being senescent, including SA-β-gal reactivity, altered expression of mediators of cell cycle checkpoints, as well as lack of the cell-cycle-associated Ki67 protein.^21^ Despite losing the ability to proliferate and migrate, senescent cells remain viable over an extended period of time and secrete a plethora of proteins known as the senescence-associated secretory phenotype (SASP), which influences the TME beneficial to tumor progression. Indeed, studies have identified premature senescence as a key player in modulating the responses to chemotherapy.^22^, ^23^ Research on breast cancer demonstrated that drug treatment triggered the generation of senescent cells from which multidrug-resistant colonies with aggressive stem-like phenotype emerged.^24^*In vivo* experiments also confirmed that after DOX administration, breast cancer developed characteristics of senescence, with the secretion of cytokines that led to the phosphorylation of signal transducer and activator of transcription 3 (STAT3), a substantial contributor to chemoresistance.^25^ In addition, colorectal mouse models suggested that late disease recurrence was associated with the senescent state in response to drug stimuli, and interruption of this state augmented the apoptotic response of colorectal cancer cells to SN38, hinting at the reversibility of chemotherapy-induced senescence.^26^ In the current study, we first ruled out the influence of multidrug resistant protein and found that collagen played a role in regulating the cell cycle to help bladder cancer cells evade apoptosis. We further demonstrated that cellular senescence, rather than dormancy, was responsible for cell cycle arrest in the presence of collagen, finally resulting in low sensitivity to chemotherapy. Meanwhile, MMC or DOX treatment caused an elevation in SA-β-Gal levels in bladder cancer cells under this 3D culture condition, which provided proof of the involvement of the senescent state in chemoresistance development. Compared to previous work, we initially addressed the relationship between chemotherapy and cellular senescence in a 3D culture model, underscoring that drug administration in conjunction with collagen culture enabled bladder cancer cells to enter a premature senescence-like state, thus impairing the curative effect of cytotoxic agents. Also, our study offered new explanations for collagen-mediated chemoresistance in bladder cancer and added further insights into the role of collagen in tumor development.

The above findings prompted us to unveil the specific molecular pathways driving cellular senescence. Senescence activation is executed by intracellular and extracellular signals and depends heavily on the engagement of the ECM.^27^, ^28^ Integrins, transmembrane receptors interacting with the matrix elements, are involved in the control of complicated cellular behaviors, including adhesion, proliferation, migration, survival, and cell fate transitions. Upon ligand binding, integrins recruit intracellular proteins and activate downstream signals, such as Ras-ERK, PI3K/AKT, and Yes1-associated transcriptional regulator (YAP)/tafazzin (TAZ) pathways.^29^ Importantly, compelling findings have established the role of integrins in cellular senescence. β1-Integrin activation was proved to be essential for tenascin-C-induced senescence of fibroblasts, which secreted soluble factors driving the malignant transformation of epithelial cells.^30^ In addition, when exposed to ionizing radiation, lung carcinoma cells might undergo a shift from cell apoptosis to premature senescence via the integrin α6β4–Src–AKT signaling pathway.^14^ Consistently, our *in vitro* and *in vivo* experiments confirmed that collagen enhanced the activation of the integrin–AKT axis to mediate a state of premature senescence in bladder cancer, leading to low reactivity to cytotoxic agents. We further identified the p53/p21 pathway, a core regulator of the cell cycle, as the downstream molecules of integrin signals. Notably, inhibitors and monoclonal antibodies of integrins have been demonstrated to dampen tumor progression and disrupt angiogenesis in mouse models.^31^ More importantly, integrin-targeted drugs (e.g. cilengitide, etaracizumab, CNTO 95) are being extensively investigated in cancer clinical trials with promising results.^32^ Both clinical and experimental data has suggested the tumor suppressive effects of cilengitide (an RGD pentapeptide ανβ3 and ανβ5 integrin inhibitor) in clinical glioblastoma,^33^ and E7820 (an integrin α2β1 inhibitor) in combination with erlotinib for non-small cell lung cancer treatment.^34^ Here, our study determined the efficacy of TFA in combination with MMC, which hindered bladder cancer growth and prolonged the survival time of tumor-bearing mice. On the basis of these results, our study illustrated the following points. (1) The expression of collagen was correlated with tumor recurrence and could be potentially translated into a prognostic marker for bladder cancer. (2) Bladder cancer cells cultured in collagen gels were skewed toward premature senescence rather than cellular apoptosis in response to chemotherapeutic agents. (3) Induction of the senescent state was dependent on the integrin–AKT axis. (4) The suppression of tumor growth could be achieved by MMC (or other chemotherapeutic agents) in cooperation with an integrin α2β1 ligand peptide, which opened up possibilities for targeting integrins in oncological treatment.

In brief, our study indicated that chemotherapy triggered premature senescence to evade apoptosis in bladder cancer cells within collagen gels. We provided evidence for the integrin–AKT axis being a regulator of senescence via the p53/p21 pathway. Targeting integrin signals may provide a feasible strategy to improve the clinical treatment for bladder cancer.

## References

[1] Bray F , Ferlay J, Soerjomataram I et al. Global cancer statistics 2018: GLOBOCAN estimates of incidence and mortality worldwide for 36 cancers in 185 countries. CA Cancer J Clin. 2018;68(6):394–424.. doi: 10.3322/caac.21492. 30207593 10.3322/caac.21492

[2] Antoni S , Ferlay J, Soerjomataram I et al. Bladder cancer incidence and mortality: A global overview and recent trends. Eur Urol. 2017;71:(1):96–108. doi: 10.1016/j.eururo.2016.06.010. 10.1016/j.eururo.2016.06.010 27370177

[3] Lenis AT , Lec PM, Chamie K et al. Bladder cancer: a review. JAMA. 2020;324(19):1980–91. doi: 10.1001/jama.2020.17598. 33201207 10.1001/jama.2020.17598

[4] Holohan C , Van Schaeybroeck S, Longley DB et al. Cancer drug resistance: an evolving paradigm. Nat Rev Cancer. 2013;13(10):714–26.. doi: 10.1038/nrc3599. 24060863 10.1038/nrc3599

[5] Gottesman MM , Fojo T, Bates SE. Multidrug resistance in cancer: role of ATP-dependent transporters. Nat Rev Cancer. 2002;2(1):48–58.. doi: 10.1038/nrc706. 11902585 10.1038/nrc706

[6] Bouwman P , Jonkers J. The effects of deregulated DNA damage signalling on cancer chemotherapy response and resistance. Nat Rev Cancer. 2012;12(9):587–98.. doi: 10.1038/nrc3342. 22918414 10.1038/nrc3342

[7] Yeldag G , Rice A, Del Río Hernández A. Chemoresistance and the Self-Maintaining Tumor Microenvironment. Cancers (Basel). 2018;10(12):471. doi: 10.3390/cancers10120471. 10.3390/cancers10120471 PMC6315745 30487436

[8] Lee SW , Park SE, Jeong GS. Sporadic cell death in macroscale 3D tumor grafts with high drug resistance by activating cell-ECM interactions. Biofabrication. 2021;13(4):045022. doi: 10.1088/1758-5090/ac24dd. 10.1088/1758-5090/ac24dd 34496353

[9] Naci D , Berrazouane S, Barabé F et al. Cell adhesion to collagen promotes leukemia resistance to doxorubicin by reducing DNA damage through the inhibition of Rac1 activation. Sci Rep. 2019;9(1):19455. doi: 10.1038/s41598-019-55934-w. 31857649 10.1038/s41598-019-55934-w PMC6923425

[10] Cooper J , Giancotti FG. Integrin signaling in cancer: mechanotransduction, stemness, epithelial plasticity, and therapeutic resistance. Cancer Cell. 2019;35(3):347–67.. doi: 10.1016/j.ccell.2019.01.007. 30889378 10.1016/j.ccell.2019.01.007 PMC6684107

[11] Baltes F , Pfeifer V, Silbermann K et al. β-Integrin binding to collagen type 1 transmits breast cancer cells into chemoresistance by activating ABC efflux transporters. Biochim Biophys Acta Mol Cell Res. 2020;1867(5):118663. doi: 10.1016/j.bbamcr.2020.118663. 31987794 10.1016/j.bbamcr.2020.118663

[12] Qiu Y , Qiu S, Deng L et al. Biomaterial 3D collagen I gel culture model: A novel approach to investigate tumorigenesis and dormancy of bladder cancer cells induced by tumor microenvironment. Biomaterials. 2020;256:120217. doi: 10.1016/j.biomaterials.2020.120217. 32736172 10.1016/j.biomaterials.2020.120217

[13] Haider T , Pandey V, Banjare N et al. Drug resistance in cancer: mechanisms and tackling strategies. Pharmacol Rep. 2020;72(5):1125–51.. doi: 10.1007/s43440-020-00138-7. 32700248 10.1007/s43440-020-00138-7

[14] Jung SH , Lee M, Park HA et al. Integrin α6β4-Src-AKT signaling induces cellular senescence by counteracting apoptosis in irradiated tumor cells and tissues. Cell Death Differ. 2019;26(2):245–59.. doi: 10.1038/s41418-018-0114-7. 29786073 10.1038/s41418-018-0114-7 PMC6329762

[15] Kumari R , Jat P. Mechanisms of cellular senescence: cell cycle arrest and senescence associated secretory phenotype. Front Cell Dev Biol. 2021;9:645593. doi: 10.3389/fcell.2021.645593. 33855023 10.3389/fcell.2021.645593 PMC8039141

[16] Senthebane DA , Rowe A, Thomford NE et al. The role of tumor microenvironment in chemoresistance: to survive, keep your enemies closer. Int J Mol Sci. 2017;18(7):1586. doi: 10.3390/ijms18071586. 10.3390/ijms18071586 PMC5536073 28754000

[17] Moon S , Ok Y, Hwang S et al. A marine collagen-based biomimetic hydrogel recapitulates cancer stem cell niche and enhances progression and chemoresistance in human ovarian cancer. Mar Drugs. 2020;18(10):1586. doi: 10.3390/md18100498. 10.3390/md18100498 PMC7599646 33003514

[18] Deng S , Li L, Xu S et al. Promotion of gastric tumor initiating cells in a 3D collagen gel culture model via YBX1/SPP1/NF-κB signaling. Cancer Cell Int. 2021;21(1):599. doi: 10.1186/s12935-021-02307-x. 34758833 10.1186/s12935-021-02307-x PMC8579534

[19] Hou L , Lin T, Wang Y et al. Collagen type 1 alpha 1 chain is a novel predictive biomarker of poor progression-free survival and chemoresistance in metastatic lung cancer. J Cancer. 2021;12(19):5723–31.. doi: 10.7150/jca.59723. 34475986 10.7150/jca.59723 PMC8408119

[20] Gonzalez LC , Ghadaouia S, Martinez A et al. Premature aging/senescence in cancer cells facing therapy: good or bad?. Biogerontology. 2016;17(1):71–87.. doi: 10.1007/s10522-015-9593-9. 26330289 10.1007/s10522-015-9593-9

[21] Lee S , Schmitt CA. The dynamic nature of senescence in cancer. Nat Cell Biol. 2019;21(1):94–101. doi: 10.1038/s41556-018-0249-2. 10.1038/s41556-018-0249-2 30602768

[22] Gewirtz DA , Holt SE, Elmore LW. Accelerated senescence: an emerging role in tumor cell response to chemotherapy and radiation. Biochem Pharmacol. 2008;76(8):947–57.. doi: 10.1016/j.bcp.2008.06.024. 18657518 10.1016/j.bcp.2008.06.024

[23] Guillon J , Petit C, Toutain B et al. Chemotherapy-induced senescence, an adaptive mechanism driving resistance and tumor heterogeneity. Cell Cycle. 2019;18(19):2385–97.. doi: 10.1080/15384101.2019.1652047. 31397193 10.1080/15384101.2019.1652047 PMC6738909

[24] Achuthan S , Santhoshkumar TR, Prabhakar J et al. Drug-induced senescence generates chemoresistant stemlike cells with low reactive oxygen species. J Biol Chem. 2011;286(43):37813–29.. doi: 10.1074/jbc.M110.200675. 21878644 10.1074/jbc.M110.200675 PMC3199523

[25] Jackson JG , Pant V, Li Q et al. p53-mediated senescence impairs the apoptotic response to chemotherapy and clinical outcome in breast cancer. Cancer Cell. 2012;21(6):793–806.. doi: 10.1016/j.ccr.2012.04.027. 22698404 10.1016/j.ccr.2012.04.027 PMC3376352

[26] Hayward RL , Macpherson JS, Cummings J et al. Antisense Bcl-xl down-regulation switches the response to topoisomerase I inhibition from senescence to apoptosis in colorectal cancer cells, enhancing global cytotoxicity. Clin Cancer Res. 2003;9(7):2856–65. 12855666

[27] Gutiérrez-Fernández A , Soria-Valles C, Osorio FG et al. Loss of MT1-MMP causes cell senescence and nuclear defects which can be reversed by retinoic acid. EMBO J. 2015;34(14):1875–88.. doi: 10.15252/embj.201490594. 25991604 10.15252/embj.201490594 PMC4547893

[28] Jun J-I , Lau LF. The matricellular protein CCN1 induces fibroblast senescence and restricts fibrosis in cutaneous wound healing. Nat Cell Biol. 2010;12(7):676–85.. doi: 10.1038/ncb2070. 20526329 10.1038/ncb2070 PMC2919364

[29] Hamidi H , Ivaska J. Every step of the way: integrins in cancer progression and metastasis. Nat Rev Cancer. 2018;18(9):533–48.. doi: 10.1038/s41568-018-0038-z. 30002479 10.1038/s41568-018-0038-z PMC6629548

[30] Fujita M , Sasada M, Eguchi M et al. Induction of cellular senescence in fibroblasts through β1-integrin activation by tenascin-C-derived peptide and its protumor effect. Am J Cancer Res. 2021;11(9):4364–79. 34659892 PMC8493383

[31] Li Z-H , Zhou Y, Ding Y-X et al. Roles of integrin in tumor development and the target inhibitors. Chin J Nat Med. 2019;17(4):241–51.. doi: 10.1016/S1875-5364(19)30028-7. 31076128 10.1016/S1875-5364(19)30028-7

[32] Łasiñska I , Mackiewicz J. Integrins as a new target for cancer treatment. Anticancer Agents Med Chem. 2019;19(5):580–6.. doi: 10.2174/1871520618666181119103413. 30451118 10.2174/1871520618666181119103413

[33] Reardon DA , Neyns B, Weller M et al. Cilengitide: an RGD pentapeptide ανβ3 and ανβ5 integrin inhibitor in development for glioblastoma and other malignancies. Future Oncol. 2011;7(3):339–54.. doi: 10.2217/fon.11.8. 21417900 10.2217/fon.11.8

[34] Ito K , Semba T, Uenaka T et al. Enhanced anti-angiogenic effect of E7820 in combination with erlotinib in epidermal growth factor receptor-tyrosine kinase inhibitor-resistant non-small-cell lung cancer xenograft models. Cancer Sci. 2014;105(8):1023–31.. doi: 10.1111/cas.12450. 24841832 10.1111/cas.12450 PMC4317852

