# Correction to: Blockade of integrin signaling reduces chemotherapy-induced premature senescence in collagen cultured bladder cancer cells

**DOI:** 10.1093/pcmedi/pbac027

**Published:** 2022-11-18

This is a correction to: Linghui Deng, Kun Jin, Xianghong Zhou, Zilong Zhang, Liming Ge, Xingyu Xiong, Xingyang Su, Di Jin, Qiming Yuan, Chichen Zhang, Yifan Li, Haochen Zhao, Qiang Wei, Lu Yang, Shi Qiu, Blockade of integrin signaling reduces chemotherapy-induced premature senescence in collagen cultured bladder cancer cells, *Precision Clinical Medicine*, Volume 5, Issue 2, June 2022, pbac007, https://doi.org/10.1093/pcmedi/pbac007

In the originally published version of this manuscript, p53/p21 pathway was erroneously referred to as p21/p53 pathway in four places.

This error has now been corrected.

